# Development of 3D PVA scaffolds for cardiac tissue engineering and cell screening applications†

**DOI:** 10.1039/c8ra08187e

**Published:** 2019-02-14

**Authors:** Elisabetta Dattola, Elvira Immacolata Parrotta, Stefania Scalise, Gerardo Perozziello, Tania Limongi, Patrizio Candeloro, Maria Laura Coluccio, Carmine Maletta, Luigi Bruno, Maria Teresa De Angelis, Gianluca Santamaria, Vincenzo Mollace, Ernesto Lamanna, Enzo Di Fabrizio, Giovanni Cuda

**Affiliations:** Department of Health Sciences, University of Magna Graecia 88100 Catanzaro Italy elisabettadattola@gmail.com +39-3202851897; Research Center for Advanced Biochemistry and Molecular Biology, Stem Cell Laboratory, Department of Experimental and Clinical Medicine, University “Magna Graecia” of Catanzaro 88100 Loc. Germaneto Catanzaro Italy; BioNEM (Bio and Nano Engineering and Technology for Medicine) Laboratory, Department of Experimental and Clinical Medicine, University of Magna Graecia 88100 Catanzaro Italy; SMILEs Lab, Physical Science and Engineering (PSE), Biological and Environmental Science and Engineering (BESE) Divisions, King Abdullah University of Science and Technology Thuwal 23955-6900 Kingdom of Saudi Arabia; DIMEG (Department of Mechanical, Energy and Management Engineering), University of Calabria 87036 Rende CS Italy

## Abstract

The aim of this study was the design of a 3D scaffold composed of poly(vinyl) alcohol (PVA) for cardiac tissue engineering (CTE) applications. The PVA scaffold was fabricated using a combination of gas foaming and freeze-drying processes that did not need any cross-linking agents. We obtained a biocompatible porous matrix with excellent mechanical properties. We measured the stress–strain curves of the PVA scaffolds and we showed that the elastic behavior is similar to that of the extracellular matrix of muscles. The SEM observations revealed that the scaffolds possess micro pores having diameters ranging from 10 μm to 370 μm that fit to the dimensions of the cells. A further purpose of this study was to test scaffolds ability to support human induced pluripotent stem cells growth and differentiation into cardiomyocytes. As the proliferation tests show, the number of live stem cells on the scaffold after 12 days was increased with respect to the initial number of cells, revealing the cytocompatibility of the substrate. In addition, the differentiated cells on the PVA scaffolds expressed anti-troponin T, a marker specific of the cardiac sarcomere. We demonstrated the ability of the cardiomyocytes to pulse within the scaffolds. In conclusion, the developed scaffold show the potential to be used as a biomaterial for CTE applications.

## Abstract 2

The aim of this study was the design of a 3D scaffold composed of poly(vinyl) alcohol (PVA) for cardiac tissue engineering (CTE) applications.

## Graphical abstract

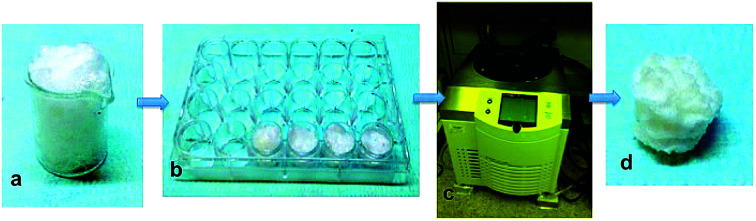

## Introduction

The recent improvements in tissue engineering^1^ allowed the creation of a wide range of three-dimensional (3D) bioengineered substitutes, which replicate natural body structures at macro, micro and nanometer resolutions.^2,3^

These matrices, called scaffolds, are a 3D temporary supports for cell growth and proliferation, and are used to regenerate damaged biological tissues or organs.^4^ A scaffold is capable of mimicking several native tissues such as lung, nerve, kidney, pancreas, heart and cardiac valves.^5–9^ Thanks to its ability to degrade in tissues, when implanted into the patient's body, scaffolds lead to the regrowth of new tissues, leaving behind a viable and purely biological system. The use of these substrates for regenerative purposes can be a valid solution for tissues and organs, which are unable to regenerate spontaneously.

The heart has a limited capability to regenerate (∼0.5% per year) after damage or disease.^10^ Therefore, these scaffolds could provide new therapeutic opportunities to treat cardiovascular diseases as an alternative to organ transplantation. The ultimate goal of cardiac tissue engineering^11–14^ is to design cardiac grafts for transplantation, which reproduce the same structure, architecture, and physiological functions of native tissues.^15^

The current trend, in most research groups, is to grow human induced pluripotent stem cells (hiPSCs) in scaffolds, and then differentiate them into cardiomyocytes to reproduce the cardiac tissue. hiPSCs are morphologically and functionally similar to human embryonic stem cells (hESCs): both are characterized by their capacity to self-renew and differentiate into all types of body cells. Despite the tremendous therapeutic potential of hESC research, their use has aroused ethical controversies since harvesting these cells involves destruction of a human embryo. The discovery of hiPSCs has overcome this crucial ethical issue since they stem from adult somatic cells such as fibroblasts and lymphocytes. hiPSCs represent powerful cell sources for tissue engineering.^16^ To date, human cardiomyocytes are easily obtained from hiPSCs through several differentiation protocols.^17^ hiPSC-derived cardiomyocytes have functional properties comparable to cardiac cells,^18^ although they retain an immature, fetal-like phenotype.^19^ 3D scaffolds can improve the efficiency in cardiomyocyte differentiation and maturation, assisting tissue growth or restoration,^20^ and represent a useful tool for a wide range of applications within cardiac tissue engineering.^21^

The ideal scaffold biomaterial for a cardiac patch is an elastic material capable of supporting thousands of stretch cycles without constraining heart contractions and relaxation.

Among synthetic biomaterials, poly(vinyl alcohol) (PVA) has a great potential for cardiac scaffold production due to its high hydrophilicity, permeability, biodegradability, biocompatibility, flexibility and capacity to be blended with other biopolymers.^22^ Therefore, it is already common in applications ranging from food to pharmaceuticals. Furthermore, poly(vinyl alcohol) is an excellent material for the production of foams and emulsions.^23^

PVA has a great capacity to retain a significant amount of water or biological fluids, swelling without dissolving. PVA is widely used in the fabrication of scaffolds due to its tunable mechanical properties: Choi *et al.*^24^ synthesized a PVA scaffold for skin regeneration by mixing gelatin and PVA. The mixture was further processed by lyophilization to obtain a porous structure. A macroporous polyvinyl alcohol scaffold was developed by Ng *et al.*^25^ to repair focal cartilage defects. This scaffold possessed characteristics suitable to support chondrocytes' growth and proliferation.

Roy *et al.*^26^ produced a 3D PVA fibrous scaffold with an electrospinning process and glutaraldehyde crosslinking. To improve the adhesion of the human mesenchymal stem cells (hMSCs) the scaffold surface was functionalized with polydopamine.

Here, we provide a hybrid method to fabricate biocompatible and biodegradable 3D porous scaffolds for cardiac tissue engineering (CTE) applications. The scaffolds were fabricated with the combination of two techniques: gas foaming and freeze-drying. We investigate how the PVA scaffolds support the hiPSCs growth and differentiation into cardiomyocytes. The proposed method of fabrication allowed obtaining different microporosities of the scaffold facilitating, at the same time, cell anchoring, nutrient diffusion, and, for *in vivo* applications, vascularization process. In addition, the proposed scaffold mimics mechanical properties close to those of the extracellular matrix (ECM) allowing cardiac cell contractility. In particular, the idea was to achieve an appropriate template to obtain cardiac cells by differentiating human induced pluripotent stem cells into cardiomyocytes.

## Methods

### Scaffold production techniques

Polymer scaffolds were manufactured by utilizing an FDA approved biodegradable PVA. In particular, a PVA solution was prepared by dissolving 8 g PVA powder (Sigma Aldrich, 99% hydrolyzed, *M*_W_ = 89 000–98 000) in 40 ml deionized water, and stirred at 80 °C until the PVA reached a final concentration of 21% w/v. After cooling it to room temperature (RT), 3 g of the obtained PVA solution was stirred with 0.66 g of sodium bicarbonate (Sigma Aldrich).

**Fig. 1:**
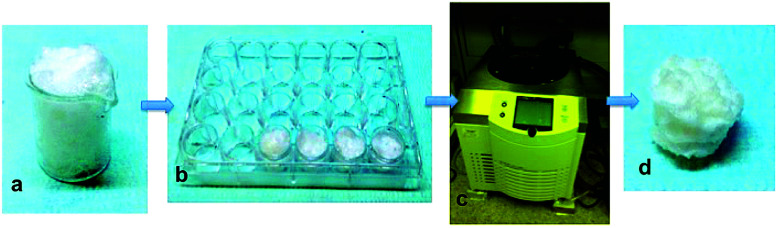
Schematic representation of the fabrication method: the polymeric foam was obtained from the gas foaming process during which the generated carbon dioxide is incorporated into the polymer (a). The PVA foam was poured in a mold (b), frozen at −20 °C for 6 h, freeze-dried for a 24 h, (c) and then the scaffolds were extracted from the mold (d).

The mixture composed of PVA and sodium bicarbonate was mixed for 30 seconds with 1 ml hydrochloric acid (HCl) (37% purity, Carlo Erba). The chemical reaction of hydrochloric acid with sodium bicarbonate particles generates carbon dioxide gas that, due to the high viscosity of the polymer, remains trapped inside of the polymer, and induces the formation of the PVA foam (Fig. 1a).^27^ The produced PVA foam was cast in a polystyrene mold (2 cm diameter) (Fig. 1b). The mold was quickly frozen at −20 °C for 6 h, to have the PVA foam stabilized (Christ alpha 1-4 lsc). The samples were freeze-dried at −20 °C under a vacuum pressure of 0.250 mbar for 24 h (Fig. 1c).^28^ The scaffold was extracted from the mold (Fig. 1d), and washed in deionized water to remove traces of unreacted components. The samples were placed to dry under a hood, and, once dried, the non-porous external surfaces of the scaffolds were cut with a surgical scalpel.

### Scanning electron microscopy (SEM) measurement

Before being analyzed, the samples were cross-sectioned using a surgical scalpel and gold-coated (∼1 nm) using a sputter coater (K650X, Quorum Technologies). Images of the scaffolds structural morphology were captured with a Dual Beam (SEM-FIB) FEI Nova 600 NanoLab system. As for the SEM acquisition, beam energy of 5 kV, and an electron current of 98 pA were used. ImageJ software (http://rsb.info.nih.gov/ij/) was used for all image analysis to assess pore size and its distribution. The measurements were carried out with the five SEM images of each sample (*n* = 5). The data are presented as mean ± S.D.

### Swelling ratio

The scaffold swelling ratio quantifies the amount of liquid material that can be absorbed. Firstly, dry scaffold (*d*) was weighed. After it was hydrated in deionized water for 24 h at RT, the wet scaffold (*w*) was weighed again. The experiment was performed in triplicate, and the swelling ratio of the scaffold was calculated using the following equation:1
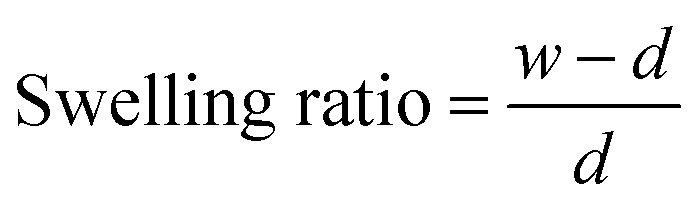
where *w* = weight of wet scaffold and *d* = weight of dry scaffold.

### Swelling kinetics of the scaffold

The swelling kinetics was estimated at room temperature (25 °C) with a gravimetric measurement using a digital microbalance. The initial dry mass of the scaffolds was recorded, and then the latter were placed in deionized water. Finally their wet weight was recorded at regular time intervals. The experiment was conducted in triplicate. The percentage swelling was calculated with the following equation:

2 S.R% = [(*w* − *d*)/*w*] × 100

where *w* = weight of wet scaffold and *d* = weight of dry scaffold.

### Stress–strain and Young modulus measurements

Uniaxial compression tests were carried out with a universal testing machine (MTS Criterion Model 42) equipped with a 100 N load cell (±0.5% accuracy). Compression platens, and an ad-hoc leak proof immersion chamber, attached to the bottom platens, were used to carry out the mechanical tests in distilled water. The tests were performed, after keeping the specimens immersed for 30 minutes, under a displacement control mode with a crosshead speed of 1 mm min^−1^. Both engineering and true stress–strain values were calculated from the force and displacement data (*F*, *δ*) obtained from the testing machine. In particular, the engineering quantities (*σ*_eng_, *ε*_eng_) were directly computed by dividing the force, and displacement data by the initial cross section area (*A*_0_), and height (*L*_0_) of the specimen, respectively:

3 *ε*_eng_ = *δ*/*L*_0_

4 *σ*_eng_ = *F*/*A*_0_

True quantities (*σ*_true_, *ε*_true_) were calculated by considering the effective area, and height of the specimen during the test, *i.e.* according to the following equations:

5 *ε*_true_ = ln(1 + *ε*_eng_)

6 *σ*_true_ = *σ*_eng_(1 + *ε*_eng_)

### Generation and characterization of human induced pluripotent stem cells (hiPSCs)

After the written informed consent and approval of the study by the ethical committee of the institution, peripheral blood mononuclear cells (PBMCs) were isolated from the whole blood of a healthy donor by a Ficoll gradient. Isolated PBMCs were treated for five days with anti-CD3 and IL-2 (both from R&D System) for T-lymphocytes activation. Reprogramming activated T-lymphocytes to their pluripotency was achieved by non-integrating the Sendai-viruses-mediated transfection of the four canonical Yamanaka's factors (*OCT4*, *SOX2*, *KLF4*, and c-*MYC*)^29^ (CytoTune™-iPS2.0 Sendai Reprogramming Kit, Life Technologies), according to the manufacturer's instructions for feeder-free iPSCs generation. Briefly, 5 × 10^5^ activated T-lymphocytes were infected at a multiplicity of infection (MOI) of 15, yielding, at d 21 of transfection, different iPSC clones. The emerged hiPSCs colonies were manually picked and transferred onto Matrigel-coated culture dishes, and cultured with mTeSR1 medium (STEMCELL Technologies, Vancouver, BC, Canada) in a humidified incubator at 37 °C at 5% CO_2_. Culture medium was changed every day. The pluripotency of generated hiPSCs was analyzed with quantitative real-time PCR (qRT-PCR) for the expression level of pluripotency-associated genes (*OCT4*, *SOX2*, *NANOG*, *c-MYC*, *KLF4* and *REX1*), and immunostaining for pluripotency markers Oct4 and Nanong. To further assess the pluripotency of the generated hiPSCs, we performed a genome-wide gene expression profile assay according to the PluriTest algorithm.^30^ Additionally, generated hiPSCs were tested for their ability to differentiate into all three germ layers by embryoid body formation assay. Markers of the three germ layers (ectoderm, mesoderm, and endoderm) on whole embryoid bodies (EBs) were tested with immunostaining and qRT-PCR. Figures relative to pluripotency assessment are shown in (ref. 31).

### Scaffold cell culture

Prior to cell seeding, scaffolds were sterilized by keeping them for 10 minutes in a 1 ml solution composed of 70% of ethanol (34 963, Sigma-Aldrich), and 30% of sterile Milli-Q water, and then sterilized under ultraviolet light for 1 night. Subsequently, scaffolds were functionalized through pre-incubation with a Matrigel solution (Corning) diluted in DMEM/F12 (Thermo Fisher Scientific) medium and, incubated at 37 °C and 5% CO_2_ for 15 min. hiPSCs were seeded onto scaffolds either as single cells (2 × 10^5^ cells/1 cm^2^ scaffold) or small clumps, and cultured in mTeSR1 medium.

### Immunofluorescence

To monitor hiPSCs' behavior inside of the PVA scaffold, after seven and twelve days since cell seeding, scaffolds were fixed with 4% paraformaldehyde for 15 min at room temperature, permeabilized (0.1% Triton, 10% FBS in DPBS), and stained with DAPI (4′-6-diamidino-2-phenylindole) for 5 min at room temperature to label cell nuclei. Stained hiPSCs onto PVA scaffolds were observed under a fluorescence microscope (Nikon Eclipse Ti) using a 405 nm excitation laser and 10× objective.

In order to detect hiPSCs-derived CMs, at day 20 of differentiation, cells were dissociated from the scaffold by treating them with 1.5 mg ml^−1^ Collagenase type II in Hank's Balanced Salt Solution (Thermo Fisher Scientific) to isolate CMs from the matrix and seeded onto fibronectin-coated Lab-Tek Chamber Slides. The day after, CMs were fixed and permeabilized as described above and treated with a blocking solution (0.1% Triton, 1% FBS in DPBS) for 2 h at 37 °C. Finally, CMs were incubated with anti-troponin T (cTNT, mouse monoclonal, Thermo Scientific) primary antibody overnight at 4 °C. After several washes with DPBS, a goat anti-mouse Alexa-Fluor-488-conjugated secondary antibody (Thermo Fisher Scientific) was added for 1 h. Following incubation with secondary antibody, cells were washed again with DPBS to remove unconjugated antibodies, while nuclei were counterstained with DAPI. Slides were mounted with fluorescent mounting medium (Dako Cytomation). Microscopy was performed using imaging system (DMi8). For each scaffold, 20 images were taken.

### SEM measurement of hiPSCs grown on PVA scaffolds

The hiPSCs were dissociated as small clumps with a gentle cell dissociation reagent (Stem Cell Technologies) and grown for 7 days on PVA Matrigel-coated scaffolds. In addition, two scaffolds without cells were prepared as control substrates: one was Matrigel-coated while the second one lacked the coating. All three scaffolds (Matrigel coated scaffold with hiPSCs, Matrigel coated scaffold and nude scaffold without cells) were subjected to the fixation procedure described below. Firstly, the 3D scaffold was washed in DPBS and then fixed for 1 h using a solution of 1.2% glutaraldehyde (G5882, Sigma), and 0.1 M sodium cacodylate at 4 °C. After fixation, the substrates were washed three times with a solution of sodium cacodylate (0.1 M, pH 7.4) for 10 min, and then treated for 1 h with a solution of 1% (v/v) osmium tetroxide (CAS #20816-12-0, 19110, Electron Microscopy Sciences) in 0.1 M sodium cacodylate. Subsequently, three washes in distilled water were performed, followed by washes of 5 min each in increasing concentrations of ethanol (30%, 50%, 70%, 80%, 90%, 96% v/v). After that, the scaffolds were washed twice with 99.9% ethanol for 15 min, followed by a gradual replacement of ethanol with the hexamethyl-disilazane (HMDS, 379212, Sigma Aldrich). To perform this step, the samples were immersed in a solution of ethanol/HMDS firstly at a ratio of 3 : 1 for 10 min; secondly at a ratio of 1 : 1 for 10 min; and finally at a ratio of 1 : 3 for 30 min. Subsequently, the samples were completely air-dried under a hood, and sputtered with gold (∼1 nm) using a sputter coater (K650X, Quorum Technologies) for the SEM visualization.

### Human induced pluripotent stem cells differentiation

iPSC-derived CMs were obtained using a PSC Cardiomyocyte Differentiation Kit (Thermo Fisher Scientific) according to the manufacturer's instructions. Briefly, iPSCs at approximately 70–85% confluence were dissociated into single cells by treating them with a gentle cell dissociation reagent and 3 × 10^5^ cells were seeded onto Matrigel-coated scaffolds and cultured with a mTeSR1 medium. In order to cardiomyocytes differentiation induction, after three days since the seeding, the mTeSR1 medium was replaced with cardiomyocyte differentiation medium A for 2 days; at d 3 of differentiation, medium A was replaced with medium B, and at d 5 cells were cultured in cardiomyocyte maintenance medium up to day 20 for cardiac maturation. Beating cells were detected at d 14 of differentiation.

### Evaluation of cardiomyocyte contractility

The development of contractile strength of hiPSC-derived CMs cultured onto the scaffold was daily monitored and observed under an optical microscope (Zeiss Axiovert 25 microscope). Cell contraction was resumed at d 14 of differentiation using a digital camera Canon Power Shot G5 (see Movie s1 in ESI†).

## Results

### SEM measurement

Cardiac scaffolds should have a highly porous structure with efficiently interconnected pores to allow the vascularization, the flow of nutrients and the elimination of waste products. The use of gas foaming technique in combination with a controlled freeze-drying process led to the formation of highly porous PVA scaffolds with characteristics suitable for CTE applications. The polymer concentration was tuned and optimized to obtain the proper foam in terms of pore size and density. In fact, the pores should have a size in the range of 80–100 μm as reported in literature,^32^ and a density that allowed interconnections among all the pores throughout the scaffold. Different concentrations of PVA were tested, and a final concentration of 21% w/v was chosen for our experiments. The produced PVA foam resulted very stable over time, and retained its shape for as long as necessary to complete the drying phase with no bubble (pores) breakdown. The CO_2_ bubbles were a template system that generated the largest pores while the freeze-drying process produced smaller pores.

**Fig. 2:**
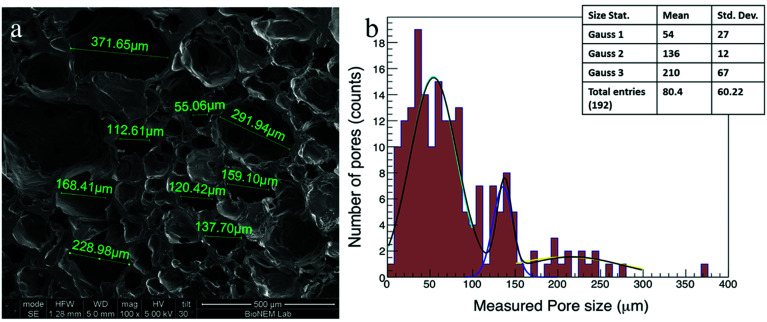
(a) The morphological characteristics of cross-sections of PVA scaffolds examined with a scanning electron microscopy (magnifications 100×, scale bar 500 μm). (b) Histograms of pore diameter distribution of the PVA scaffolds. The PVA scaffolds show an average pore size of 82 ± 60 μm. The data distribution is recapitulated from three Gaussians with the following means: 54 ± 27 μm, 136 ± 12 μm, 210 ± 67 μm. This pore distribution was achieved thanks to the two processes: gas foaming that generated largest pores, while the freeze-drying process produced smaller pores.

The SEM image (Fig. 2a) shows the scaffold morphology: the PVA scaffolds mainly had an irregular porous structure, and the pores diameter ranged from 10 μm to 370 μm with an average diameter of 82 μm ± 60 μm. In addition, the graph of pores size distribution (Fig. 2b) shows that 95% of pores in the PVA scaffold had a diameter less than 200 μm.

It is possible to represent the pore size distribution with three Gaussians^33–35^ with the following means: 54 ± 27 μm, 136 ± 12 μm, 210 ± 67 μm, where the pore size distribution with the lower mean corresponds to the pore created by the freeze-drying process.

The scaffold morphology was similar between the different samples demonstrating that the process of scaffold production has a high reproducibility. The pore size of scaffolds measured in this study was suitable for the dimension of hiPSCs. In fact, the smaller pores (<50 microns) guarantee optimal hiPSCs adhesion; pores with dimension around 100 microns provide space for cell expansion, while the largest pores (>200 microns) allow nutrients and oxygen to be delivered to cells located throughout the 3D scaffold and, for *in vivo* applications, space for vascularization. From a qualitative point of view, the pores were interconnected in all samples.

### Swelling ratio

The ability of a scaffold to preserve water is an important feature for maintaining a correct hydration state and the diffusion of some solute molecules. The swelling ratio is defined as the fractional increase in the weight of the hydrogel due to water absorption.

In general, the swelling of the scaffolds occurs in two steps. First, the water penetrates into the scaffold without distorting the structure, and secondly the elastic deformation of the structure allows the absorption of an additional water amount.

**Fig. 3:**
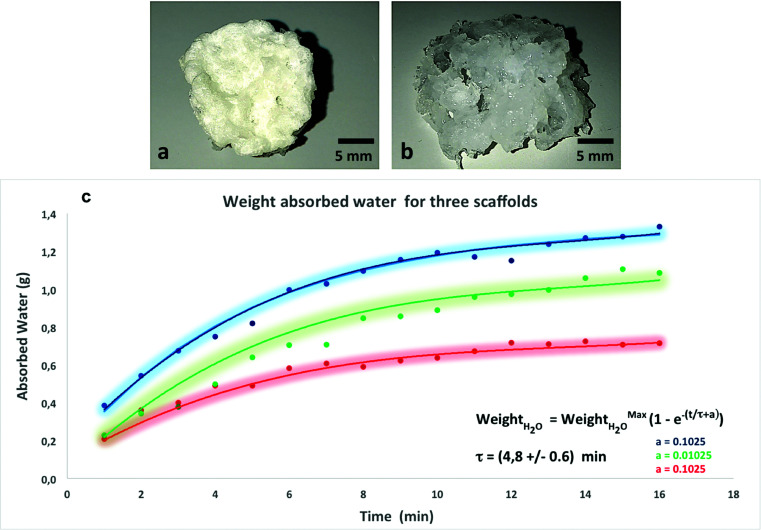
Macroscopic view in dry (a) and wet (b) conditions of PVA scaffold at room temperature. The wet condition was obtained by immersing the scaffolds for 24 hours in water. After hydration in water the scaffolds retained their three-dimensional structure. (c) Graph of water adsorption speed of the three PVA scaffolds. The points are the experimental data, while the curves are the data fit. For curve fitting of experimental data (points) the law shown in the figure was used. The time constant was 4.8 ± 0.6 min. The colored region, in penumbras, represents the trend around the curves in ±1 sigma.

The PVA scaffolds absorbed a quantity of water about 7 times greater than their dry weight maintaining their three-dimensional structure (Fig. 3).

### Swelling kinetics of the scaffold

The proteins present within the ECM such as proteoglycans are responsible for the swelling pressure of connective tissue preserving the hydration of the ECM. In order to simulate this property, the scaffold for CTE applications should have a high capacity of water absorption and retention preventing the loss of body fluid.

To evaluate the ability of a PVA scaffold to retain water, we performed a test for water uptake capacity. As shown in Fig. 3, the water uptake in PVA scaffolds gradually increased as an exponential function of time. The weight of the scaffold increases over time due to its absorption of the water inside, and finally reaches the balance when no further weight gain is recorded, which means that the material absorbed the maximum amount of water. In particular, the sponge swelled up to 400% of its original weight in 8 min, and reached equilibrium in 14 min. The three curves (Fig. 3) represent the water absorption speed of three different scaffolds. The initial values were different because the dry weight of each scaffold was in turn different and consequently their starting content of water varies. The time constant was the average of the three values (measured for each time) with the maximum error, and it was fitted with the law shown in Fig. 3. The fit of the experimental points, shown in the graph (Fig. 3) with a continuous line for the three scaffolds, had an exponential trend with an average time constant of 4.8 ± 0.6 min. The colored region, in penumbras, represents the trend around the curves in ±1 sigma.

The spread of the points around the central trend is due to experimental measurement uncertainty. The reproducibility of the measurements was carried out by testing three samples fabricated at different times.

### Stress–strain curves and Young modulus

**Fig. 4:**
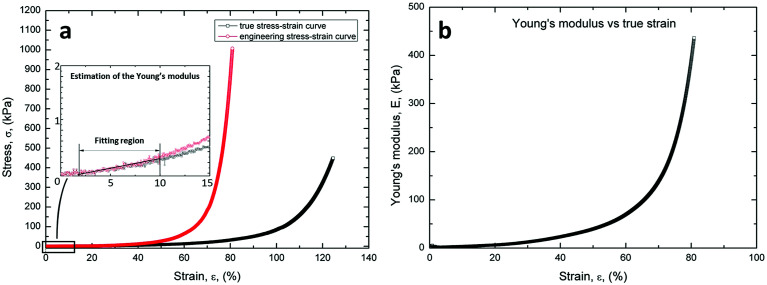
(a) Stress–strain curves of the PVA scaffold obtained from compression test. (b) Evolution of the Young's modulus as a function of true strain. The values of the Young's modulus were calculated from the true stress–strain data. The graph shows marked non-linear trend of the Young's modulus.

As expected, the specimens exhibit a marked non-linear trend of the stress–strain curve, as shown in Fig. 4a. In addition, the figure illustrates the linear fitting method used to calculate the Young's modulus.

Fig. 4b illustrates the marked non-linear trend of the Young's modulus in the whole strain range (0–80%). However, the values of the Young's modulus between the strain ranges of 2–10% and 5–10% were calculated from the true stress–strain data, according to Wang *et al.*^36^

These results were consistent with the stress–strain and Young's modulus curves measured for natural ECM. In fact, natural structures exhibit a quite low stiffness in the early stages of loading, *i.e.* large strains are observed with small load increments, and subsequent marked continuous stiffening with further increasing the compression load.

### iPSCs growth and differentiation capability on PVA scaffolds

**Fig. 5:**
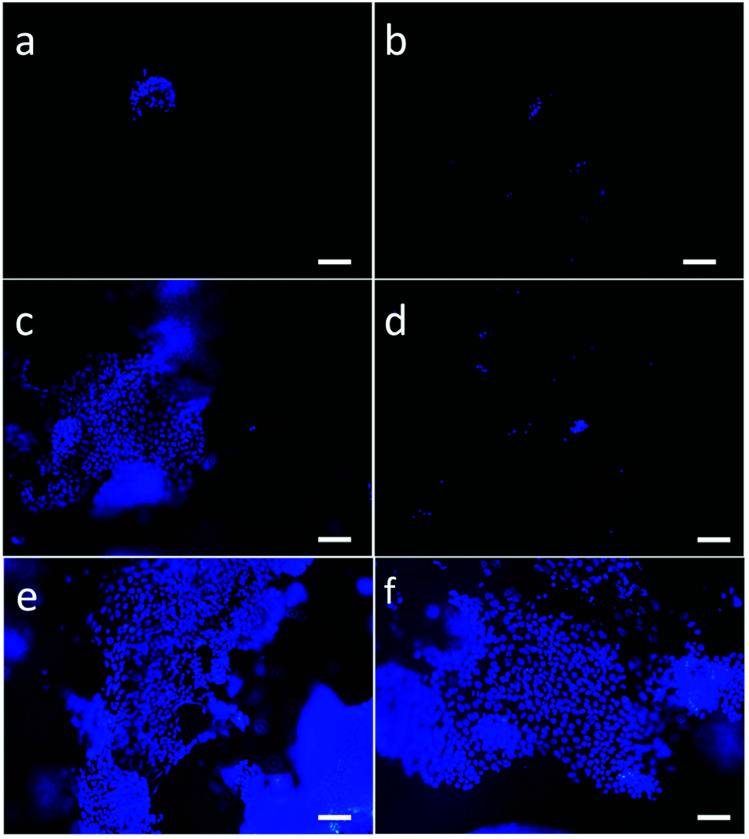
*In vitro* hiPSCs on PVA scaffolds after 7 days (a–d) and 12 days (e and f). The nuclei (blue) were stained with DAPI and observed with a 10× objective, scale bars: 100 μm. (a) Single cells on the Matrigel coated PVA scaffold. (b) Single cells on the PVA scaffold without Matrigel coating. (c) Clumps on the Matrigel coated PVA scaffold. (d) Clumps on the PVA scaffold without Matrigel coating. (e and f) hiPSCs clumps on the Matrigel coated PVA scaffold.

The potential biocompatibility of PVA scaffolds was evaluated by culturing hiPSCs, either as single cells or clumps, directly on nude or Matrigel-coated scaffolds. DAPI staining was used to evaluate the cell attachment and viability inside of the scaffold over time (Fig. 5).

hiPSCs seeded as single cells or clumps onto nude scaffolds were unable to adhere (Fig. 5b and d), compared to cells seeded on Matrigel-coated scaffolds (Fig. 5a and c).

hiPSCs seeded as small clumps onto Matrigel-coated scaffolds were chosen since this methods was the best culture condition showing a homogeneous distribution inside of the scaffold (Fig. 5c, e and f) and a good rate of proliferation. Moreover, cross-sections cut through the centers of the PVA scaffolds showed that the cells also reached the inner part of the substrate. Furthermore, we noticed a higher cell density at the edges of the scaffolds probably due to a static growth condition that generates a gradient of nutrients. Therefore, for chemotaxis the cells move to more nutrient-enhancing areas. To overcome diffusional limitations of mass transfer during cell culture, the scaffolds could be inserted in a microfluidic system that allows continuous and uniform distribution of gases and nutrients.

**Fig. 6:**
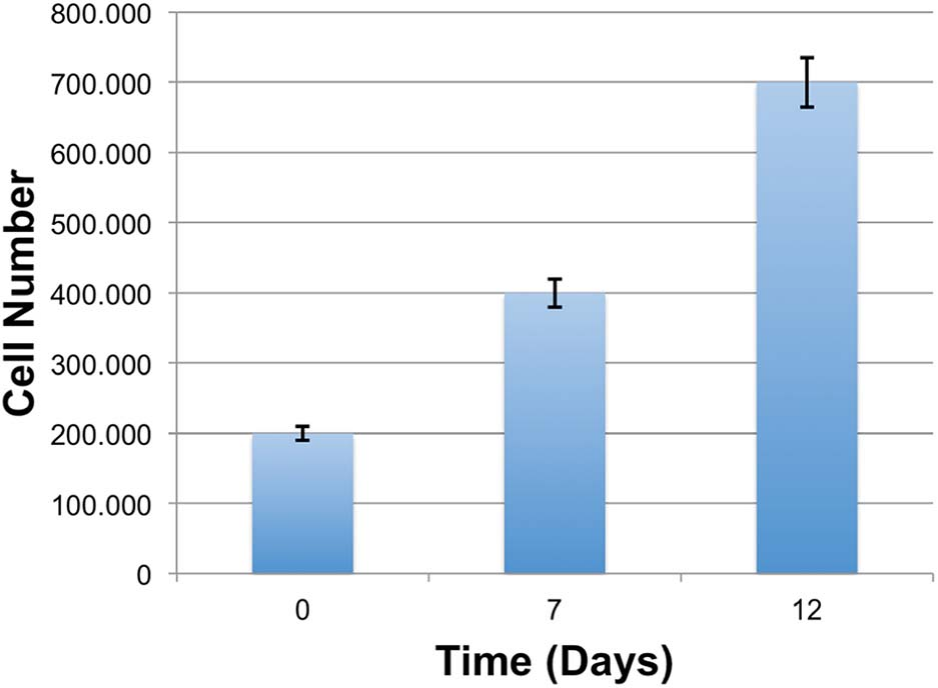
Proliferation of hiPSCs on PVA scaffolds.

To determine the growth rate of hiPSCs, we quantified cells at d 7 and d 12 of culture using ImageJ software. As shown in the chart (Fig. 6), the number of live cells on the scaffold after 7 and 12 days were progressively increased compared to the initial number of cells (d 0). This finding highlights the capacity of the scaffolds to maintain cell viability, and its ability to sustain cell adhesion and proliferation, revealing a great cytocompatibility of the PVA substrates.

The morphology and organization of hiPSCs grown on PVA scaffolds was assessed using a SEM.

The achievement of a suitable contractile performance of the artificial tissue required a high cell density, as shown in the SEM images below.

**Fig. 7:**
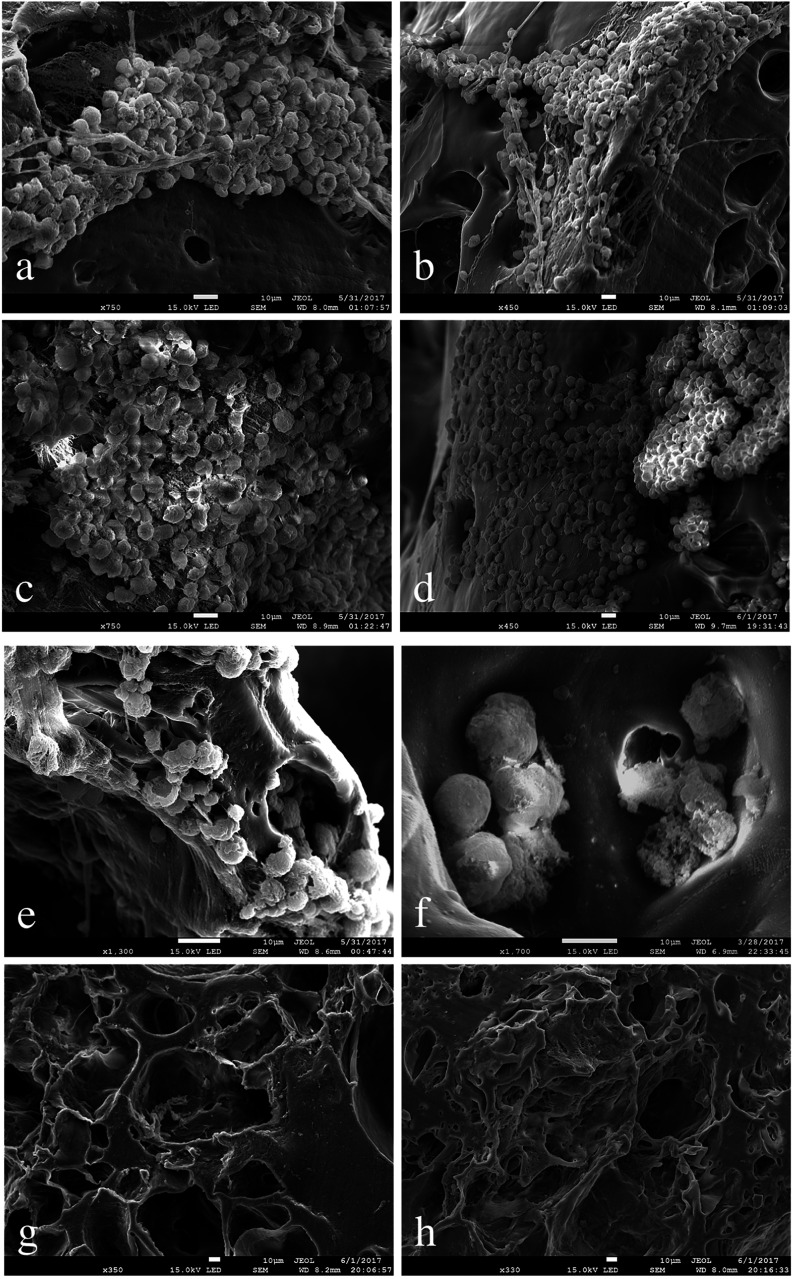
The SEM images of hiPSCs grown for 7 days on PVA scaffold shows the cells distribution on the sample: (a) colony formed by several layers; (b) colony formed by single and several layers; (c) monolayer colony; (d) single cells and colonies. Detail of the hiPSCs on the PVA scaffold (e). High resolution image of the cells grown inside of the pores (f). PVA scaffolds without Matrigel (g) and with Matrigel-coating (h) were used as control substrates. Scale bars: 10 μm.

Fig. 7 shows a uniform and homogenous distribution of cells on the substrate and a phenotypically rounded morphology of the cells seeded over time in culture (7 days).

According to the tendency of hiPSCs to grow as colonies, in some parts of the scaffolds the cells tend to organize as clumps. In Fig. 7(a and c) hiPSCs are represented as organized in a monolayer and overlapping layers, respectively. On a smaller scale, the SEM images show how the hiPSCs cells penetrated into the pores of the scaffold (Fig. 7f). hiPSCs appear small sized (around 15 μm) with a regular shape (Fig. 7f), meaning that the PVA scaffolds offer appropriate mechanical support and stability to the hiPSCs culture. Scaffolds with and without Matrigel and with no seeded hiPSCs were used in our study as negative control substrates (Fig. 7g and h).

**Fig. 8:**
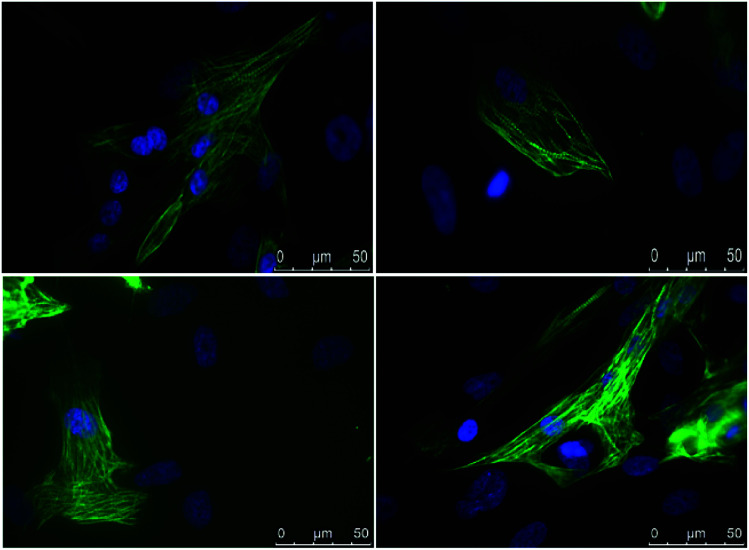
Immunostaining of cTNT (in green) in hiPSC-derived cardiomyocytes. Nuclei are stained with DAPI (in blue). Scale bar, 50 μm.

hiPSC-derived CMs were mobilized from the scaffold by treatment with collagenase and subsequently subjected to immunostaining for cTNT (a marker specific of the cardiac sarcomere) in order to confirm that differentiation towards cardiac lineage correctly took place (Fig. 8). Moreover, short *in vivo* video registration of the scaffold was performed, demonstrating the presence of beating areas within the scaffold matrix. Contractile motion is an indicator of cardiomyocytes health and maturation. Synchronous macroscopic contractions of tissue with a rate of 40–50 beats per minute were observed (see ESI: Movie 1†) around d 14 of cardiac differentiation.

The high elasticity of the scaffolds allows a normal contraction of hiPSC-derived CMs, which is the evidence of electrophysiological and mechanical properties of the engineered porous biocompatible 3D scaffold.

## Discussion

The heart is an organ with a poor regenerative capacity^10^ and the interruption of oxygen supply following ischemic event causes cardiomyocytes' death.

To date, the only therapeutic solutions for cardiac dysfunctions are drugs and/or heart transplantation.^37–39^ Based on that, new treatments are needed for treating cardiovascular diseases, that represents the worldwide leading cause of deaths.

The field of cardiac tissue engineering^1,11,12^ can provide useful strategies to regenerate the myocardium after failure or injury. The 3D scaffolds provide a better approximation of heart extracellular matrix than the conventional 2D culture systems. We focused on the design of 3D PVA scaffolds for cardiac tissue engineering showing how highly porous matrices could be simply fabricated from PVA with gas foaming in combination of freeze-drying process. PVA is a biocompatible and biodegradable polymer approved by the Food and Drug administration to produce medical devices. In this study, PVA foam was cross-linked with a freeze-drying method without using any cross-linking agents, and the absence of toxic chemicals, greatly improves the scaffold's biocompatibility.

The most significant aspect in designing the porous scaffolds is that the scaffold pore size fits to the diameter of cells. This protocol was designed and optimized to obtain a scaffold with different porosity and interconnected pores.

The well interconnected pore network contained both micro (50 μm in diameter) and macropores (200 μm in diameter). As shown by Salem *et al.*^40^ while the pore size less than 60 μm is needed to facilitate penetration of endothelial cells and their colonization of the substrate, the larger pores allow nutrient diffusion throughout the scaffold.

More in detail, a cardiac scaffold should reproduce both the architecture and the function of ECM, and to verify this property, swelling ratio and swelling kinetics of PVA scaffolds were examined.

The PVA scaffolds absorb a quantity of water about 7 times greater than their dry weight, but maintain their three-dimensional structure. Swelling studies showed that the PVA scaffold had an excellent hydrophilicity. In fact, as shown in Fig. 3, the water uptake in PVA scaffold gradually increased in function of time.

The stress–strain (Fig. 4a) and the Young modulus (Fig. 4b) of the PVA scaffolds were measured. The graphs show that the scaffolds have an elastic behavior, similar to extracellular matrix.

A further purpose of this study was to assess the biocompatibility of the PVA scaffolds with the growth and proliferation of hiPSCs and the subsequent differentiation in cardiomyocytes.

The scaffold offers the solid protection for the hiPSCs and provides an appropriate environment for cells differentiation. After 7 days of culture, the Matrigel-coated scaffolds seeded with cell groups showed more adhered cells (Fig. 5c) than other scaffolds.

Matrigel-coated scaffolds appeared to be the best condition favoring cell proliferation when hiPSCs are seeded as clumps, making the covering procedure essential for cell adherence, growth and differentiation. Seven days after seeding, the number of cells on the scaffold was doubled with respect to the initial number of plated cells, while the optimal density required for hiPSCs differentiation was reached after 12 days. Moreover, as shown by SEM analysis of the scaffolds, hiPSCs morphology proved to be well maintained and the cells were able to populate even the pores located more in depth of the 3D structure.

Previous studies have shown that 3D scaffolds with a characteristic architecture can improve the efficiency of cardiomyocytes' differentiation and maturation.^20^ Based on this statement, we investigated the PVA scaffold's capacity to affect hiPSCs-derived CMs' maturation. Our data show the efficiency of the scaffold to host hiPSCs and their capacity to sustain hiPSCs' differentiation as shown by immunostaining for cTNT and spontaneous cell contraction with a rate of 40–50 beats per min. The scaffold supported these contractions due to its high elasticity. This last aspect is crucial since CMs' contraction and their mechanical force need to be supported for a future application of a 3D scaffold in cardiac regenerative medicine. We showed that the seeded hiPSCs migrated, proliferated, and differentiated *in situ* into cardiomyocytes.

## Conclusions

Here, we show the development of biocompatible PVA porous scaffolds that have the potential to be used as a biomaterial in cardiac tissue engineering applications thanks to their high cytocompatibility.

Although our model of cardiac tissue does not perfectly mimic the mature myocardium, it can reproduce certain characteristics of the ECM such as its 3D structure and mechanical properties. We demonstrated that PVA scaffolds have the potential to promote the maturation of CMs from hiPSCs although all the developed scaffolds represent a poor approximation of the real cardiac tissue.

In this context, our 3D scaffolds represent a useful tool for a wide range of applications within cardiac tissue engineering, ranging from cardiac patches to replace cells lost through infarction, (reducing the need for organ replacement) to faster and cheaper candidates to be used as preclinical models, alternative to animal models, to test potential therapeutic compounds.

Future studies will be performed by our group to determine an effective strategy to easily quantify the number, behavior and distribution of hiPSCs cultured on 3D scaffolds. Moreover, to better characterize the differentiation potential of hiPSCs into cardiomyocyte lineage inside the scaffold, we will generate transgenic hiPSC line expressing an enhanced Green Fluorescent Protein (eGFP) under the control of a cardiac-specific gene promoter (such as cardiac troponin, *cTNT*, or α-Myosin heavy chain, *αMHC*), that will allow the hiPSC-derived cardiomyocytes' lineage to be trace directly inside of the scaffolds without the need to enzymatically mobilize CMs from the scaffold.

Finally, the iPSCs technology, together with their potential to differentiate into all cell types, represent a powerful strategy for personalized medicine since hiPSCs are derived from patient's own cells. The combination of hiPSCs potential and 3D scaffolds engineering opens the way for a new hope in the field of regenerative medicine.

## Author contributions

E. D. developed the PVA scaffold, designed and performed experiments, analyzed data and prepared the manuscript. E. I. P., S. S., M. T. D., G. S. carried out all the hiPSCs growth and differentiation into PVA scaffolds and performed biological analysis on the samples.

M. C., T. L. contributed to the fabrication process and to the analyzed data. C. M. and L. B. performed scaffolds mechanical testing. G. P., P. C., V. M., E. D. F., E. L., G. C. conceived the idea and supervised the work. All authors discussed the results and finalized the manuscript.

## Conflicts of interest

There are no conflicts to declare.

## Abbreviations

SEM: Scanning electron microscopy
HCl: Hydrochloric acid
PVA: Poly(vinyl alcohol)
CTE: Cardiac tissue engineering
EBs: Embryoid bodies
MOI: Multiplicity of infection
2D: Two-dimensional
3D: Three-dimensional
4D: Four-dimensional
iPSCs: Induced pluripotent stem cells
hiPSCs: Human induced pluripotent stem cells
hPSCs: Human pluripotent stem cells
ESCs: Embryonic stem cells
hESCs: Human embryonic stem cells
ICM: Inner cell mass
CMs: Cardiomyocytes
ECM: Extracellular matrix
hMSCs: Human mesenchymal stem cells

## Supplementary Material

RA-009-C8RA08187E-s001
